# Occupational hypersensitivity pneumonitis in salmon processing worker: novel antigenic proteins from salmon

**DOI:** 10.1093/occmed/kqag020

**Published:** 2026-04-28

**Authors:** Miriam Grgic, Marte E Holman, Berit E Bang

**Affiliations:** Department of Occupational and Environmental Medicine, University Hospital of North Norway, Postboks 16, 9038 Tromsø, Norway; Department of Occupational and Environmental Medicine, University Hospital of North Norway, Postboks 16, 9038 Tromsø, Norway; Department of Occupational and Environmental Medicine, University Hospital of North Norway, Postboks 16, 9038 Tromsø, Norway

## Abstract

To our knowledge, this is the first published case study of a salmon processing worker with occupational hypersensitivity pneumonitis, where the causative antigen was investigated by immunoblot analysis utilising the patient’s serum. Both known and previously unidentified salmon antigens were detected.

## Teaser

This is the first reported case of a rare lung inflammation in a salmon‑processing worker where the potential trigger was identified using the patient’s blood. By applying immunoblot analysis, we found that the worker’s immune system reacted not only to known salmon proteins but also to previously unidentified proteins. These findings broaden our understanding of which salmon components are relevant in the development of lung inflammation and may help improve diagnosis and workplace protection in the industry.

Key learning points **What is already know about this subject:**

- Hypersensitivity Pneumonitis (HP) is a heterogenous disease, caused by an immunologic reaction to an inhaled antigen.
- Only one case of occupational HP due to exposure at a salmon processing factory has previously been published.
- To date, there is no consensus regarding the optimal diagnostic approach; a particular issue is the lack of standardisation of serum IgG testing against potential antigens associated with HP.

**What this study adds:**

- This is the first published case report of a salmon processing worker with occupational HP where the causative antigen was investigated using immunoblot analysis of the patient’s serum.
- We detected IgG, and IgE antibodies from the patient’s serum binding to both known and unknown salmon antigens.
- This indicates the existence of new relevant occupational antigens from salmon.

**What impact this may have on practice, policy or procedure:**

- Development of an IgG HP panel could help physicians understand which exposure that is the cause of the immunologic reaction.
- This is essential in guiding patients effectively and avoid overly restrictive behaviours in the workplace that are not medically necessary.

## Background

Hypersensitivity pneumonitis (HP) is a heterogenous disease caused by an immunologic reaction to an inhaled antigen [1–3]. To date, there is no consensus regarding the optimal diagnostic approach [4]. Its diagnosis may rely on a combination of factors, including exposure history, characteristic CT scan appearance, bronchoalevolar lavage, differential cell count with or without supportive histology, and demonstration of sensitisation to known antigen by specific immunoglobulin G (IgG) [1]. Fish processing workers are exposed to bioaerosols containing fish-derived proteins, including known and unknown allergens and enzymes, as well as microorganisms and endotoxins [5–8]. Combined with cold and wet conditions in the work environment, exposure to these factors is associated with respiratory symptoms and occupational asthma reported in several previous publications [9–12]. To our knowledge, only one case of occupational HP due to exposure at a salmon processing factory has previously been published [13]. In the referred case the identification of the possible causative exposure was established using a specific inhalation challenge [13]. In this report we present a case of a salmon processing worker with occupational HP as the primary diagnosis. The aim of this study was to determine the patient’s IgG- and/or IgE-binding profile with respect to salmon proteins by using the immunoblot method.

## Case presentation

A 30-year-old female smoker, with no previous history of respiratory disease, but with known atopy, was hospitalised due to a six-months history of subacute onset dyspnoea and dry cough, that progressed gradually.

As part of the diagnostic evaluation in the months prior to hospitalisation, a CT scan was performed after the GP’s referral. The CT showed diffuse ground-glass opacities in all lung lobes, raising the question of bronchopneumonia or interstitial lung disease. Following this, the GP attempted treatment with courses of antibiotics, but only with transient improvement. After a while, it became apparent to the GP that the patient showed a work-related symptom pattern, with clear worsening after returning to work at a salmon processing factory. On further assessment of the temporal relationship between her symptoms and occupational exposure, it became evident that a few weeks after starting to work as a salmon processing worker, she had developed lower respiratory symptoms at work, and later experienced red swollen eyes during working hours. She described a classic pattern of improvement of symptoms in periods off work, and increased symptoms after returning to work. Pulmonary function testing was performed and revealed moderate obstruction. Bronchodilator response test showed an FEV_1_ increase of 15% of the predicted value consistent with asthma. The patient was started on inhaled corticosteroids but reported no improvement and subsequently discontinued the medication on her own.

On admission, arterial blood gas analysis showed reduced pO_2_ at 9.8 kPa, with SaO_2_ at 95%. pCO_2_ was 4.2 kPa. She had mildly elevated leukocytes at 12.2 × 10^9^/L, and C-Reactive Protein (CRP) was negative. Airway polymerase chain reaction (PCR) was normal. The patient was admitted to the ward with supplemental oxygen via mask as needed and for further evaluation. The main suspicion was interstitial lung disease. Extended blood tests were performed to assess connective tissue disease or other underlying causes. A new CT scan showed partial regression of ground-glass opacities in the upper and middle lobes. New opacities had appeared in both lower lobes, with a combination of ground-glass and a ‘crazy paving’ pattern. It was noted that the patient had peripheral blood eosinophilia at 2.3 × 10^9^/L. Extended blood tests showed elevated total IgE at 310 kU/L (reference value <120 kU/L), as well as a markedly increased IgE-salmon at 18 kU/L (reference value <0.35 kU/L). Aspergillus specific IgE, ANA/ANCA screening, and other rheumatologic tests were otherwise negative. The patient experienced clinical improvement without treatment during the 4-day hospital stay, was not oxygen-dependent, and reported less dyspnoea. Imaging findings with typical interstitial changes, combined with exposure to known antigen and improvement on avoidance of antigen exposure was considered highly suggestive of occupational HP, which was supported later by presence of IgG-salmon in blood sample (12 mg/L (no reference values)). At discharge, the patient was prescribed prednisolone 30 mg once daily for four weeks. After being diagnosed she avoided further exposure to salmon bioaerosols and was referred to our occupational medicine outpatient clinic. She got an appointment a few weeks after she was diagnosed with occupational HP. At our clinic, the patient was in good physically health, with no respiratory symptoms.

## Investigations

Patient serum was collected at Department of Laboratory Medicine at University Hospital Northern Norway following standard procedures. IgG and IgE binding to proteins in extracts from salmon tissues were tested using immunoblot assays [14, 15]. Extracts of salmon tissues were prepared in our laboratory following established protocols [14].

## Results

**Figure 1.**
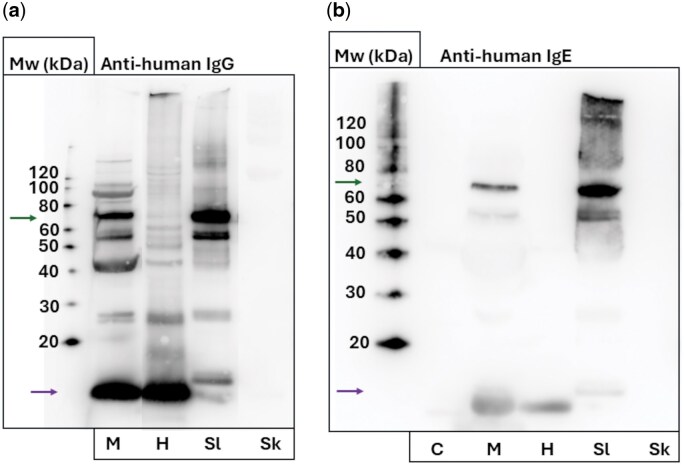
Immunoblots with patient serum and anti-human IgG and anti-human IgE antibodies against extracts from different salmon tissues. (a) IgG antibody binding MW—molecular weight marker (Magic mark, Sigma Aldrich), M—muscle extract, H—heat treated muscle extract, Sl—outer mucus extract (slime layer), Sk—skin extract. 70 kDa region is marked with a green arrow, and the 12 kDa (parvalbumin) region is marked with a purple arrow. In raw muscle extract IgG exhibited the strongest affinity for proteins in the 12 kDa region and proteins in the 40–70 kDa area. In heat-treated muscle extracts, IgG bound to proteins in 12 kDa region and to proteins within 40–70 kDa region, although with a weaker signal. In mucus extract, the strongest IgG signal was observed for proteins in the 50–70 kDa range. (b) IgE antibody binding, MW—molecular weight marker (Magic mark, Sigma Aldrich), C—positive control (cod parvalbumin, ALK), M—muscle extract, H—heat treated muscle extract, Sl—outer mucus extract (slime layer), Sk—skin extract. In raw muscle extract there was weak binding to proteins in 12 kDa region, and within the 50–70 kDa region, there was stronger binding to the 70 kDa band. In heat treated muscle extract, IgE bound only in ∼12 kDa region. In mucus extract binding in the same region (50–70 kDa) was observed.

As seen in Figure 1a, IgG demonstrated strong binding in the ∼12 kDa molecular weight area and multiple higher molecular weight proteins within the 40–70 kDa range in both raw muscle and heat-treated muscle extracts, though the intensity was reduced in the heat-treated samples. In Figure 1b, IgE showed prominent binding to proteins within the 50–70 kDa range in the raw muscle extract, but no corresponding signal was observed in the heat‑treated muscle sample within this region. Notably, both IgG and IgE exhibited robust binding to proteins in the 50–70 kDa range in the mucus extract. Neither IgG nor IgE bound to proteins from the salmon skin extract, which contained only collagen.

## Discussion

We report the case of a woman employed in a salmon processing factory who developed progressively worsening dyspnoea and a dry cough after the onset of her employment, demonstrating a clear work‑related pattern of symptoms. Her symptoms were consistent with HP, and exposure to the suspected causative antigen was confirmed by serological testing demonstrating the presence of salmon-specific IgG, in addition to imaging findings compatible with interstitial lung disease. Based on this, she was diagnosed with occupational HP. Blood tests also showed sensitisation to salmon with an IgE‑mediated response. Her clinical history included episodes of red, swollen eyes and respiratory symptoms occurring at work, and pulmonary function tests demonstrated moderate reversible obstruction—findings highly suggestive of allergic asthma. However, the lack of clinical improvement on inhaled corticosteroids, together with CT imaging that favoured HP over asthma, reduced the likelihood of asthma as the primary diagnosis. Nevertheless, a dual diagnosis cannot be entirely excluded, as it remains possible that the patient developed both allergic asthma and HP after beginning her employment at the salmon processing factory.

To our knowledge, this is the first published case report of a salmon processing worker with occupational HP where the causative antigen was investigated using immunoblot analysis of the patient’s serum. As part of the immunoblotting protocol, proteins are separated by molecular weight, thereby enabling more precise detection of the proteins that may act as causative antigens. Previous research has demonstrated that fish and other seafood allergens are present in inhalable aerosols sampled from the workers breathing zone, demonstrating that airway exposure is probably the main exposure route in this type of work environment [5, 6, 8, 10]. According to published data, concentrations of specific salmon antigens generally fall within the low to mid ng/m³ range, while total inhalable protein levels are generally in the µg/m³. In the patient’s serum we detected IgG, and IgE antibodies that were binding to both known and unknown salmon antigens. In muscle extract, both immunoglobulins bound to proteins at the ∼12 kDa size range, corresponding to size of the known salmon antigen parvalbumin [16]. In muscle extract, IgG additionally bound to a protein in the 40 kDa range. This is the binding region of the known IgE-reactive salmon protein aldolase [17]. In the IgE blot, only faint binding in that region could be observed in the muscle extract. Most interestingly, in both muscle and mucus extracts, both antibodies bound to proteins in the 50–70 kDa range. In heat treated muscle extract, IgE bound only in ∼12 kDa range, implying that IgE-binding proteins in the 50–70 kDa range are heat sensitive. In a recent publication, several of the potential antigens in this region have been identified [15]. However, further research is needed to identify their clinical relevance in disease development. At present, a key challenge is the lack of standardisation in serum IgG assays targeting potential antigens associated with HP [4]. In up to 60% of patients with HP, the specific antigen and exposure source remain unidentified [4]. The presented case highlights the need for development of IgG HP panel featuring specific antigens [4]. Identification of the specific causative antigen is important, as antigen avoidance is the treatment cornerstone of symptomatic HP disease. Such diagnostics tool would enable clinicians to accurately identify the exposure that is the cause of the immunologic reaction and provide better patient guidance. This approach is essential in minimising restrictive behaviours in the workplace that are not medically necessary.

## References

[1] Kongsupon N , Walters GI, Sadhra SS. Occupational causes of hypersensitivity pneumonitis: a systematic review and compendium. Occup Med (Lond) 2021;71:255–259. 34370035 10.1093/occmed/kqab082 PMC8486273

[2] Nogueira R , Melo N, Novais E Bastos H *et al*. Hypersensitivity pneumonitis: antigen diversity and disease implications. Pulmonology 2019;25:97–108. 30126802 10.1016/j.pulmoe.2018.07.003

[3] Zhang X , Igor B, Elena D, Olga R, Glazachev O. Prevalence of occupational hypersensitivity pneumonitis: a systematic review and meta-analysis. Int J Environ Health Res 2024;34:3891–3908. 38544315 10.1080/09603123.2024.2333021

[4] Raghu G , Remy-Jardin M, Ryerson CJ *et al*. Diagnosis of hypersensitivity pneumonitis in adults. An official ATS/JRS/ALAT clinical practice guideline. Am J Respir Crit Care Med 2020;202:e36–e69. 32706311 10.1164/rccm.202005-2032ST PMC7397797

[5] Shiryaeva O , Aasmoe L, Straume B *et al*. Respiratory effects of bioaerosols: exposure-response study among salmon-processing workers. Am J Ind Med 2014;57:276–285. 24310925 10.1002/ajim.22281

[6] Bonlokke JH , Bang B, Aasmoe L *et al*. Exposures and health effects of bioaerosols in seafood processing workers—a position statement. J Agromedicine 2019;24:441–448. 31453763 10.1080/1059924X.2019.1646685 PMC9048166

[7] Madsen AM , Thomassen MR, Frederiksen MW *et al*. Airborne bacterial and fungal species in workstations of salmon processing plants. Sci Total Environ 2024;951:175471. 39137839 10.1016/j.scitotenv.2024.175471

[8] Thomassen MR , Hollund BE, Özgümüs T *et al*. Occupational exposure to bioaerosols in the Norwegian salmon processing industry. Ann Work Expo Health 2025;69:708–721. 40583266 10.1093/annweh/wxaf038 PMC12313455

[9] Jeebhay MF , Cartier A. Seafood workers and respiratory disease: an update. Curr Opin Allergy Clin Immunol 2010;10:104–113. 20179585 10.1097/ACI.0b013e3283373bd0

[10] Dahlman-Höglund A , Renström A, Larsson PH, Elsayed S, Andersson E. Salmon allergen exposure, occupational asthma, and respiratory symptoms among salmon processing workers. Am J Ind Med 2012;55:624–630. 22576678 10.1002/ajim.22067

[11] Lopata AL , Jeebhay MF. Airborne seafood allergens as a cause of occupational allergy and asthma. Curr Allergy Asthma Rep 2013;13:288–297. 23575656 10.1007/s11882-013-0347-y

[12] Fagernæs CF , Lauritzen HB, Tøndell A *et al*. Occupational asthma in the salmon processing industry: a case series. Occup Environ Med 2024;81:400–406. 39137970 10.1136/oemed-2024-109564

[13] Tjalvin G , Svanes Ø, Bertelsen RJ *et al*. Hypersensitivity pneumonitis in fish processing workers diagnosed by inhalation challenge. ERJ Open Res 2018;4:00071–2018. 10.1183/23120541.00071-2018 PMC6168762 30302334

[14] Elda I , Grgic M, Stensvåg K, Bang B. Tissue-based skin prick test extracts from Atlantic salmon containing occupationally relevant allergens. Front Allergy 2025;6:1525012. 40626259 10.3389/falgy.2025.1525012 PMC12230974

[15] Elda I , Grgic M, Tjalvin G *et al*. Sensitization to salmon among occupationally exposed Norwegian salmon processing workers: Identification of IgE-reactive proteins. Front Allergy 2026;7:1735903. In press. 41777728 10.3389/falgy.2026.1735903 PMC12950587

[16] Kuehn A , Swoboda I, Arumugam K, Hilger C, Hentges F. Fish allergens at a glance: variable allergenicity of parvalbumins, the major fish allergens. Front Immunol 2014;5:179. 24795722 10.3389/fimmu.2014.00179 PMC4001008

[17] Kuehn A , Hilger C, Lehners-Weber C *et al*. Identification of enolases and aldolases as important fish allergens in cod, salmon and tuna: component resolved diagnosis using parvalbumin and the new allergens. Clin Exp Allergy 2013;43:811–822. 23786287 10.1111/cea.12117

